# Beyond Risk Scores: Current and Emerging Strategies for Cardiotoxicity Risk Prediction in Cardio-Oncology

**DOI:** 10.3390/cancers18203260

**Published:** 2026-10-09

**Authors:** Benedetta Volpi, Daniela Cardinale

**Affiliations:** Cardioncology Unit, European Institute of Oncology, IRCCS, 20141 Milan, Italy

**Keywords:** cardiotoxicity, risk prediction, artificial intelligence

## Abstract

**Background:** Cardiotoxicity remains a major limitation of contemporary cancer therapies, affecting morbidity and long-term outcomes among cancer survivors. Early identification of patients at increased cardiovascular risk is essential to guide surveillance and preventive strategies. **Methods and Results:** We performed a narrative review of currently available risk assessment and prediction approaches for cancer therapy-related cardiotoxicity, including consensus-based frameworks, statistically derived prediction models, and emerging machine learning-based approaches. Widely used tools, such as the HFA-ICOS (Heart Failure Association–International Cardio-Oncology Society) risk assessment framework, provide a practical and standardized approach to baseline risk stratification and are increasingly implemented in clinical practice. However, their discriminative performance appears variable across studies, and their applicability across diverse oncologic populations may be limited. Key challenges include heterogeneity in cardiotoxicity definitions, limited external validation, and incomplete integration of dynamic parameters. Overall, models based primarily on clinical variables demonstrate only moderate predictive accuracy, particularly in patients receiving anthracyclines. **Conclusions:** Current risk stratification strategies provide a useful foundation for clinical decision-making but remain limited in their ability to fully capture individual risk. Future approaches should integrate clinical variables with circulating biomarkers, advanced imaging, and AI to enable more dynamic and individualized risk prediction in cardio-oncology.

## Simple Summary

Cardiotoxicity is a major complication of modern cancer therapies and can significantly impact long-term outcomes in cancer patients. Identifying individuals at higher cardiovascular risk before and during treatment is essential to guide monitoring and preventive strategies. Several risk assessment and prediction approaches have been developed, ranging from consensus-based clinical frameworks to statistically derived and more advanced tools incorporating imaging, biomarkers, and artificial intelligence (AI). However, these approaches show variable performance and applicability across different patient populations. This review summarizes existing risk stratification tools, highlights their limitations, and discusses emerging approaches that may improve personalized prediction of cardiotoxicity in oncology.

## 1. Introduction

Cardiotoxicity represents a major limitation of modern cancer therapies and is increasingly recognized as an important determinant of long-term morbidity and survival among cancer patients. With the increasing use of potentially cardiotoxic treatments, including anthracyclines, targeted therapies, immune checkpoint inhibitors, and newer anticancer agents, cardiovascular complications have emerged as a critical challenge in contemporary oncology care [1].

Accurate identification of patients at high cardiovascular risk is essential to guide surveillance strategies, optimize preventive interventions, and minimize treatment interruptions. Early recognition of cancer therapy-related cardiovascular toxicity may allow timely cardioprotective management while preserving oncologic efficacy [2].

Conventional cardiovascular risk prediction models often perform suboptimally in patients with cancer because they were largely derived from non-oncologic populations and do not account for competing cancer-related mortality, treatment-specific toxicities, or the complex bidirectional interactions between malignancy, anticancer therapies, and cardiovascular disease [3].

**Figure 1:**
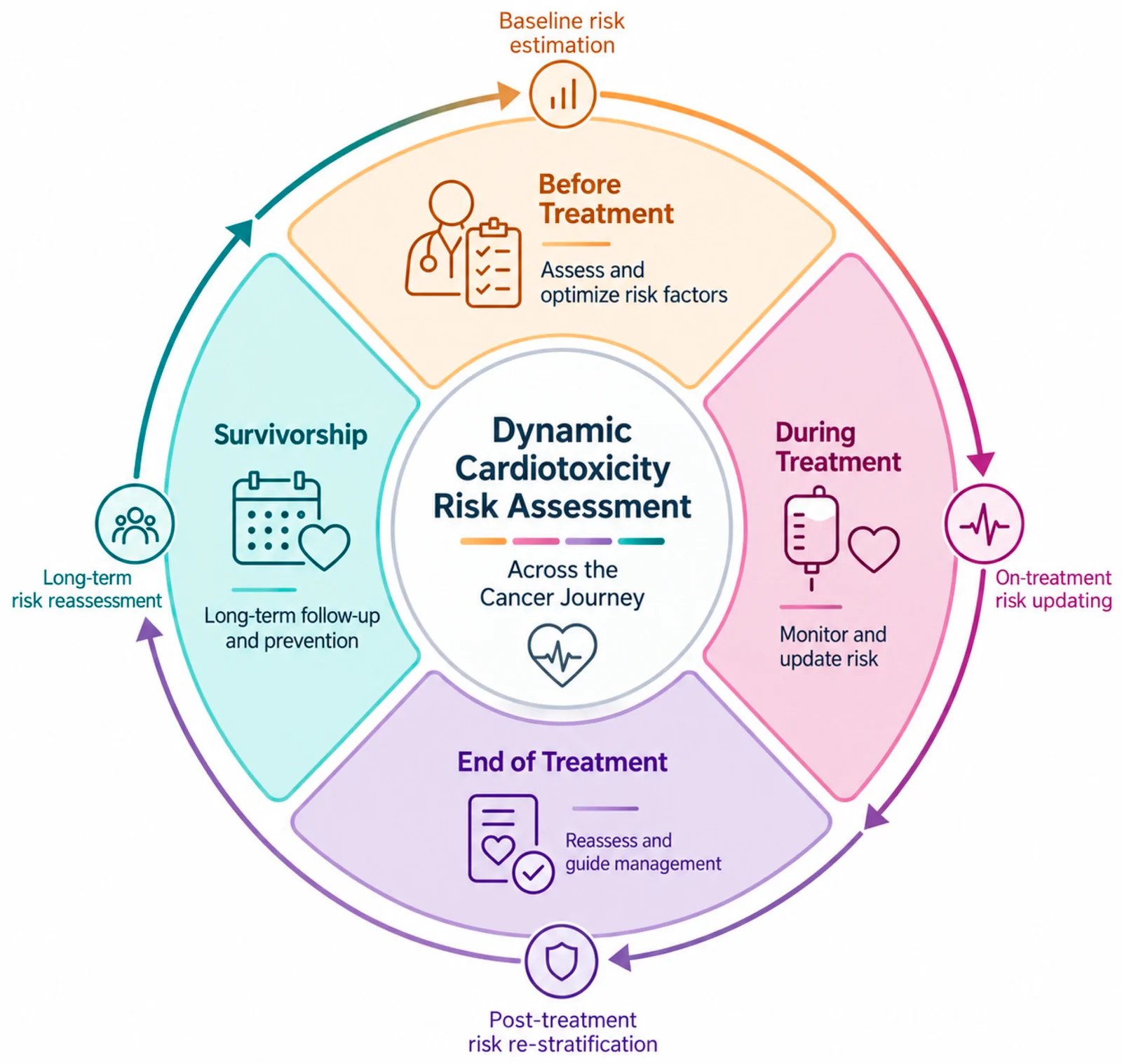
Dynamic cardiotoxicity risk prediction across the cancer journey.

Predicting cardiovascular toxicity in cardio-oncology poses unique challenges compared with other cardiovascular settings. Unlike traditional cardiovascular risk factors, which generally evolve over years, the risk of cardiotoxicity is highly dynamic. It may change substantially throughout the cancer journey according to tumour type and stage, cumulative treatment exposure, modifications in therapeutic strategy, treatment response, and patient-specific characteristics (Figure 1). Consequently, effective risk stratification requires a longitudinal and adaptive approach, with reassessment at multiple time points before, during, and after cancer therapy rather than relying exclusively on baseline risk estimation.

Over the past decade, several approaches have been developed to support cardiovascular risk assessment and prediction in oncology patients [4]. These range from consensus-based clinical frameworks to statistically derived prediction models and emerging multimodal and computational approaches. Among them, the HFA-ICOS risk assessment framework has emerged as the most widely adopted tool in clinical practice, providing a structured approach to baseline cardiovascular risk stratification across different cancer therapies [5].

Despite these advances, robust evidence supporting their clinical implementation remains limited [4]. Importantly, available approaches address substantially different cardiovascular outcomes—including cancer therapy-related cardiac dysfunction (CTRCD), heart failure, major adverse cardiovascular events (MACE), or cardiovascular mortality—making direct comparisons challenging and contributing to marked heterogeneity in reported performance. Furthermore, only a minority of statistically derived models have undergone robust external validation, frequently in selected patient populations, and available validation studies are often heterogeneous with respect to cancer type, treatment exposure, outcome definitions, and follow-up duration [6]. As a result, the generalizability and clinical reliability of many currently available models remain uncertain.

Additional limitations include inconsistent definitions of cardiotoxicity, variable calibration across populations, limited incorporation of longitudinal data, and insufficient personalization of risk estimation [3]. These limitations suggest that further improvement in risk prediction is unlikely to be achieved using static clinical variables alone. Consequently, increasing attention is being directed toward multimodal approaches integrating circulating biomarkers, advanced imaging techniques, genomic and omics data, and AI-based methodologies to enable more precise and individualized risk prediction [7]. Importantly, most currently available cardiotoxicity risk-prediction tools have been developed in populations receiving anthracyclines and/or HER2-targeted therapies, and their applicability to newer anticancer treatments with distinct cardiovascular toxicity phenotypes remains uncertain.

This narrative review summarizes established and emerging approaches to cardiotoxicity risk prediction in oncology, with particular emphasis on clinically implemented risk assessment approaches, externally validated models, and novel strategies integrating imaging, biomarkers, and AI.

### Literature Search Strategy and Study Selection

This narrative review was informed by a structured literature search of PubMed/MEDLINE, Embase, and Scopus (inception to 20 August 2026), combining terms related to cardio-oncology and cardiotoxicity (“cardio-oncology”, “cardiotoxicity”, “CTRCD”) with terms on risk prediction (“risk score”, “prediction model”, “risk stratification”) and emerging approaches (“biomarker”, “echocardiography”, “cardiac magnetic resonance”, “artificial intelligence”, “machine learning”, “digital twin”). Reference lists of key reviews, guidelines, and included articles were also hand-searched.

Titles/abstracts and full texts were independently screened by two authors, with disagreements resolved by discussion. We included original studies on the development, validation, or application of cardiotoxicity risk-prediction tools, along with relevant systematic reviews, meta-analyses, and guidelines, prioritizing studies reporting derivation cohort, external validation, and discrimination/calibration data; case reports, conference abstracts, and non-peer-reviewed sources were excluded. Only English-language publications were considered.

Consistent with the narrative scope of this review, study selection followed a traceable but non-systematic process based on clinical relevance and methodological quality, rather than a formal PRISMA-based systematic approach.

## 2. Consensus-Based Risk Assessment Frameworks

Early risk prediction approaches developed to predict cardiotoxicity in patients with cancer primarily relied on expert consensus and simple clinical parameters rather than formal statistical modelling. One of the earliest examples is the Mayo Clinic Cardiotoxicity Risk Score, which combines patient-related characteristics—including prior cardiovascular disease, traditional cardiovascular risk factors, previous exposure to radiation therapy or anthracyclines, age, and sex—with treatment-related factors reflecting the intrinsic cardiotoxic potential of administered therapies [8]. Notably, this score was developed based on expert consensus and existing evidence instead of being derived from a dedicated development cohort, which may limit its predictive performance compared with data-driven models [9].

More recently, the HFA-ICOS risk assessment framework has emerged as the most widely adopted consensus-based tool in contemporary cardio-oncology practice [5]. Developed collaboratively by the Heart Failure Association of the European Society of Cardiology and the International Cardio-Oncology Society, the HFA-ICOS risk assessment tool provides a structured framework for baseline cardiovascular risk stratification across different cancer therapies. Separate treatment-specific proformas incorporate pre-existing cardiovascular disease, baseline cardiac function, circulating cardiac biomarkers (when available), demographic characteristics, cardiovascular risk factors, prior exposure to cardiotoxic therapies, and lifestyle variables. Weighted scoring is then used to classify patients into low-, moderate-, high-, or very high-risk categories.

From a methodological perspective, HFA-ICOS proformas were not derived from a formal development cohort and generate categorical rather than continuous quantitative risk estimates, and do not predict a single standardized endpoint, as clinically relevant cardiovascular outcomes vary according to the anticancer therapy considered. Rather, they function as a structured clinical risk assessment framework, providing actionable categories that guide clinical decision-making. Low-risk patients can typically proceed with anticancer treatment without delay, while high-risk patients may require closer surveillance, multidisciplinary evaluation, or consideration of treatment modification. Finally, intermediate-risk cases rely on expert clinical judgment.

The HFA-ICOS framework is also the most frequently externally evaluated cardio-oncology risk tool to date. The majority of studies has been conducted in adult patients with breast cancer, whereas evidence in other malignancies and treatment settings remains more limited [4]. Its prognostic value was supported by Rivero-Santana et al. in the CARDIOTOX (CARDIOvascular TOXicity induced by cancer-related therapies) registry (*n* = 1066), where increasing HFA-ICOS risk categories were significantly associated with both cardiotoxicity and all-cause mortality, with good discriminative performance (AUC 0.78 at 1 year and 0.76 at 2 years) [10]. Similarly, Di Lisi et al. supported its risk-stratification value in a prospective cohort of breast cancer patients receiving anthracycline-based regimens, with or without trastuzumab [11].

However, not all validation studies have yielded consistent results. For example, Tini et al. found no significant association between HFA-ICOS categories and cardiotoxicity risk, possibly due to treatment-related confounding (e.g., more cardioactive therapy at baseline in high-risk patients) [12]. Several factors may explain these discrepant findings, including differences in treatment allocation, patient characteristics, and endpoint definitions; however, the underlying reasons remain incompletely understood. Interpretation of available validation studies is limited by substantial heterogeneity in cardiotoxicity definitions, which range from overt heart failure and clinically significant CTRCD to milder subclinical imaging or biomarker abnormalities [13].

In addition, although HFA-ICOS has demonstrated reasonable discriminative ability in several cohorts, its calibration across diverse populations remains insufficiently characterized. Some studies have suggested that observed event rates may exceed those anticipated by the assigned risk categories, particularly when broader definitions of CTRCD are adopted [4].

Overall, current evidence supports the HFA-ICOS tool as the most practical framework for baseline cardiovascular risk assessment in cardio-oncology. However, its ability to provide accurate individualized risk prediction remains limited, underscoring the need for robust, data-driven, and dynamically updated prediction models.

## 3. Statistical Prediction Models

To overcome the limitations of consensus-based frameworks, several statistical prediction models have been developed using large observational cohorts and formal multivariable modelling techniques. Unlike consensus-based frameworks, these models are formally derived from patient-level data and are intended to estimate outcome probabilities within a defined population and prediction horizon. Their validity therefore depends not only on discrimination, but also on calibration, adequate event numbers, and performance in independent populations.

One of the first statistically derived models, proposed by Ezaz et al., was developed and validated in a large Surveillance, Epidemiology, and End Results (SEER)-Medicare cohort of older women with early-stage breast cancer treated with adjuvant trastuzumab [14]. This point-based model incorporates readily available clinical variables—including age, treatment type, and cardiovascular comorbidities—and stratifies patients into distinct risk categories. The incidence of heart failure or cardiomyopathy increased markedly across these categories, demonstrating the model’s ability to discriminate risk using routine clinical data.

Building on this approach, Abdel-Qadir et al. developed a broader cardiovascular risk prediction model in a population-based cohort of over 90,000 women with early-stage breast cancer [15]. Unlike previous models focusing primarily on heart failure, this score predicts major adverse cardiovascular events—including heart failure, ischemic events, and cardiovascular death—while accounting for competing risks. Incorporating routinely available clinical variables such as age and pre-existing comorbidities, the model allows estimation of both 5- and 10-year cardiovascular risk, with patients in the highest risk categories exhibiting markedly increased event rates compared with low-risk individuals.

Further refining long-term prediction, Barac et al. developed a 10-year heart failure/cardiomyopathy risk model in a longitudinal cohort of 26,044 women with newly diagnosed invasive local or regional breast cancer [16]. By integrating baseline demographics, cardiovascular comorbidities, cancer stage, and treatment exposures—including anthracyclines, HER2-targeted therapy, radiation, and endocrine treatment—the model demonstrated good calibration and a time-dependent AUC of 0.79 at 10 years. Patients could be stratified into low-, intermediate-, and high-risk groups with observed 10-year event rates of 1.7%, 6.3%, and 19.4%, respectively.

While these models represent a substantial methodological advance over consensus-based frameworks, they remain largely static, relying on baseline variables. To address this gap, the RE-ACT (REassessment of Anthracycline Cardiotoxicity Risk after Therapy) score was recently developed to refine risk prediction after anthracycline completion [17]. Derived in 2612 adult cancer patients and externally validated in 819 patients, RE-ACT incorporates only two post-treatment variables: left ventricular ejection fraction (LVEF) and cumulative anthracycline dose. Despite its simplicity, the model demonstrated promising discriminatory performance (AUC 0.83 in the derivation cohort and 0.88 in the validation cohort), outperforming HFA-ICOS alone. Importantly, its post-chemotherapy application shifts risk assessment toward the survivorship phase, when long-term cardiovascular surveillance can be more effectively tailored. Its very high negative predictive value (97–99%) may be particularly useful for identifying patients at sufficiently low risk to avoid unnecessary prolonged cardiac monitoring. Rather than replacing baseline assessment, RE-ACT therefore complements HFA-ICOS within a two-step strategy, linking pretreatment risk stratification with a pragmatic post-treatment reassessment that may help define subsequent surveillance intensity.

## 4. Imaging and Biomarkers for Risk Prediction

### 4.1. Echocardiography

Echocardiography remains the most widely used and accessible imaging modality in cardio-oncology, offering the ability to perform serial, non-invasive assessments of cardiac function and risk stratification in patients undergoing potentially cardiotoxic therapies. Current practice guidelines recognize echocardiography as central to baseline and longitudinal risk assessment [18] as well as to cardiotoxicity detection [13].

Among echocardiographic parameters, global longitudinal strain (GLS) has emerged as a robust early predictor of CTRCD and future cardiotoxic events, often detecting subclinical myocardial impairment before changes in LVEF occur [19]. Baseline GLS and its early reductions during therapy have been associated with a higher likelihood of subsequent cardiotoxicity, providing risk stratification beyond conventional measures such as LVEF [20,21].

Beyond GLS, emerging evidence suggests that other echocardiographic markers may provide additional prognostic information. Left atrial strain parameters, particularly peak atrial longitudinal strain and left atrial reservoir strain, have been associated with the subsequent development of CTRCD and may identify atrial dysfunction before overt changes in LVEF occur [22]. Similarly, abnormalities in right ventricular (RV) function, including RV strain, have been linked to adverse cardiovascular outcomes in cancer patients [23]. However, evidence is less extensive than for GLS, and further studies are needed to validate these parameters across diverse cancer cohorts, and to integrate these parameters in clinical scores.

Importantly, echocardiography enables dynamic assessment of risk through serial measurements taken before, during, and after cancer therapy. Tracking changes in strain or other deformation indices allows clinicians to identify individuals whose risk trajectory is increasing, potentially prompting earlier cardioprotective interventions or treatment modification [24]. Such serial evaluation captures temporal changes in myocardial function that static baseline models cannot.

Despite its strengths, echocardiographic risk prediction faces challenges related to inter-vendor variability and limited standardization of strain measurements, which can affect reproducibility and external comparability; efforts by expert societies to harmonize strain acquisition and reporting are ongoing to improve consistency across centers [25].

### 4.2. Cardiac Magnetic Resonance

Beyond echocardiography, cardiac magnetic resonance (CMR) has emerged as a promising imaging modality for cardiotoxicity risk stratification by providing highly reproducible assessment of ventricular function together with detailed characterization of myocardial mechanics and tissue composition. Although most CMR studies have focused on the early detection of CTRCD, interest is growing in the identification of baseline imaging markers that may predict susceptibility to cardiotoxicity before overt myocardial injury occurs [26]. However, evidence supporting the prognostic role of baseline tissue characterization parameters, such as native T1 mapping, extracellular volume fraction, or late gadolinium enhancement, remains limited and inconsistent [27].

Among baseline CMR variables, strain-derived parameters have yielded the most promising results. In a prospective study, Giusca et al. demonstrated that CMR-derived strain parameters predicted subsequent cardiotoxicity more accurately than conventional functional indices, with the percentage of preserved myocardial segments emerging as a potential early risk marker [28]. Similarly, the PRoactive Evaluation of Function to Evade Cardio Toxicity (PREFECT) study evaluated 59 patients with breast cancer or lymphoma undergoing anthracycline-based chemotherapy [29]. Among baseline imaging parameters, the CMR-derived MyoHealth score, a segmental strain-based index reflecting preserved myocardial deformation, demonstrated the highest predictive performance, outperforming both baseline CMR LVEF and 2D echocardiographic LVEF. Baseline circumferential strain parameters were also associated with subsequent CTRCD.

Although these results are promising, several limitations must be emphasized. Both studies were small and single-center, limiting generalizability. Follow-up durations were relatively short, endpoints varied in definition, and the cohorts primarily included anthracycline-treated breast cancer patients, restricting applicability to other therapies or malignancies. Consequently, while CMR strain and tissue markers may provide potential incremental value for baseline risk prediction, these findings require validation in larger, multicenter, and more heterogeneous populations. Furthermore, it remains uncertain whether CMR-derived strain metrics provide clinically meaningful incremental predictive value beyond widely available echocardiographic measures such as GLS.

### 4.3. Cardiac Computed Tomography

Cardiac computed tomography (CT) currently has a limited but evolving role in predicting cardiotoxicity. Evidence to date focuses primarily on the coronary artery calcium (CAC) score, which reflects subclinical atherosclerotic burden and may help identify patients at higher risk of future cardiac events during cancer therapy [30].

In the ANTEC (Atherosclerosis iN chemoTherapy-rElated Cardiotoxicity) study, prospective evaluation of moderate- and high-risk patients receiving anthracycline-based chemotherapy demonstrated that a CAC score greater than zero was associated with a significantly higher incidence of subsequent CTRCD, independent of traditional clinical risk factors [31]. This study highlights the potential utility of CAC measured on baseline CT scans to stratify cardiovascular risk before chemotherapy initiation.

Despite these encouraging findings, the use of cardiac CT for risk prediction remains exploratory, with evidence limited to small cohorts and retrospective analyses.

### 4.4. Biomarkers

#### 4.4.1. Myocardial Injury and Hemodynamic Stress

Circulating biomarkers have emerged as valuable tools for cardiovascular surveillance and risk refinement in patients undergoing potentially cardiotoxic cancer therapies. Established cardiac biomarkers, particularly cardiac troponins and natriuretic peptides, reflect objective measures of myocardial injury and hemodynamic stress and are widely incorporated into contemporary cardio-oncology guidelines for baseline evaluation and treatment surveillance. Their assessment is recommended in selected patient populations, reflecting their ability to identify patients with underlying cardiovascular vulnerability and to provide a reference point for subsequent treatment-related changes [13].

However, the role of biomarkers in predicting cardiotoxicity before treatment initiation remains incompletely defined. Although elevated baseline troponin or natriuretic peptide levels have been associated with an increased risk of subsequent cardiovascular events in some studies, findings have been inconsistent across different populations, treatment regimens, and definitions of cardiotoxicity [32,33,34,35]. Consequently, current evidence suggests that these biomarkers may be more useful for longitudinal monitoring and early detection of evolving myocardial injury than for providing robust standalone estimates of future cardiotoxic risk [36].

#### 4.4.2. Inflammation and Endothelial Dysfunction

Interest has increasingly extended to biomarkers reflecting biological pathways beyond direct cardiomyocyte injury, including inflammation, angiogenesis, and endothelial activation [37,38]. In a prospective study of patients with HER2-positive breast cancer receiving anthracycline- and trastuzumab-based treatment, pretreatment concentrations of angiopoietin-2, endoglin, endothelin-1, and myeloperoxidase were more strongly associated with subsequent CTRCD than conventional cardiac biomarkers, which did not differ significantly between patients who did and did not develop cardiac dysfunction [39].

#### 4.4.3. Vascular and Hypertensive Toxicity with Anti-VEGF Agents

Distinct biomarker categories have emerged for therapy classes whose principal cardiovascular mechanism is vascular rather than myocardial. In patients treated with anti-VEGF tyrosine kinase inhibitors and bevacizumab, baseline systolic blood pressure has been identified as a predictor of subsequent treatment-induced hypertension [40]. Circulating and germline biomarkers have also shown predictive value: lower baseline plasma levels of VEGF-A and angiopoietin-2 have been associated with a higher risk of bevacizumab-induced hypertension [41], while germline polymorphisms in VEGFA and, more robustly, the KCNAB1 variant rs6770663, identified through genome-wide association analysis and independently replicated, have been linked to differential hypertension risk across multiple bevacizumab-treated cohorts [42,43]. These preliminary findings suggest that, for at least some anti-angiogenic agents such as bevacizumab, candidate predictive biomarkers may be vascular and pharmacogenomic in nature rather than indicators of direct cardiomyocyte injury, although evidence remains limited and has not been consistently replicated across all agents in this class.

#### 4.4.4. Immune-Mediated Toxicity with Checkpoint Inhibitors

Immune checkpoint inhibitors (ICIs) present a different challenge, as myocarditis is an immune-mediated complication that may develop early after treatment initiation. Although cardiac troponins have an established role in the diagnostic evaluation of suspected myocarditis and may be used for surveillance in selected patients, their ability to identify susceptible individuals before treatment remains uncertain [13]. A retrospective case–control study identified a baseline hematological profile associated with subsequent ICI-associated myocarditis, but independent external validation is lacking [44]. Similarly, preliminary autoantibody findings have been reported in a very small pilot study [45]. Current evidence in this setting therefore remains exploratory, and these candidate biomarkers cannot yet support routine pretreatment risk stratification.

Overall, biomarker-based strategies provide important mechanistic insights and may enhance risk stratification when integrated with clinical characteristics, therapy class, and imaging findings. At present, however, no single circulating biomarker has demonstrated sufficient predictive performance to serve as a standalone tool for baseline cardiotoxicity risk assessment across all cancer therapies; rather, the choice of biomarker should be guided by the specific treatment-related mechanism of cardiovascular injury anticipated.

Beyond circulating protein and haematological biomarkers, germline genetic variants and multi-omics signatures represent a complementary layer of individualized risk prediction, spanning anthracycline metabolism pathways, oxidative stress genes, and transcriptomic markers; these approaches are discussed in detail in Section 5.3.

## 5. Emerging Prediction Strategies: Artificial Intelligence and Beyond

### 5.1. Machine Learning and Deep Learning Approaches

**Figure 2:**
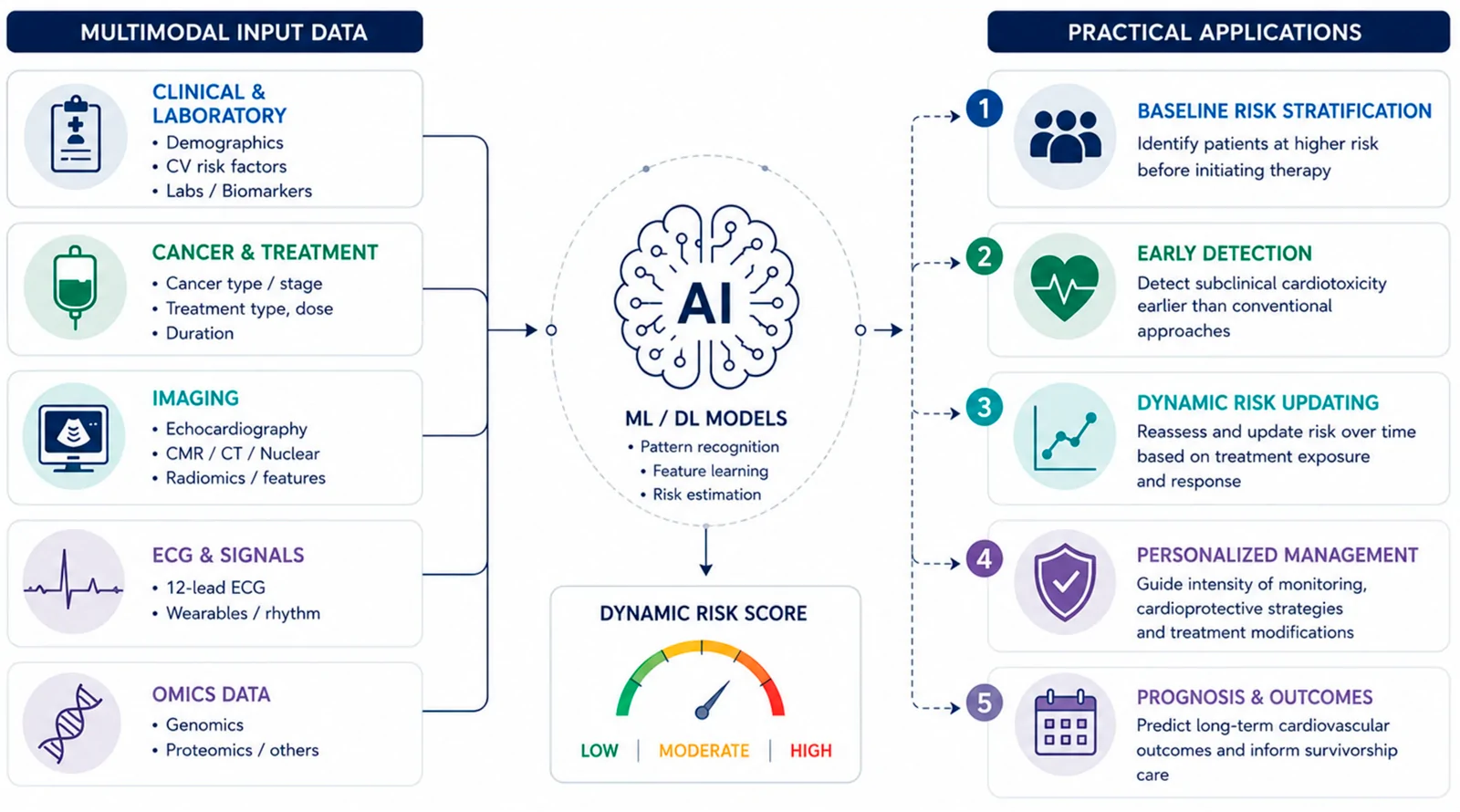
Overview of emerging AI applications in cardiotoxicity risk prediction. AI, artificial intelligence; CV, cardiovascular; CMR, cardiovascular magnetic resonance; CT, computed tomography; ECG, electrocardiography; ML, machine learning.

Machine learning (ML) and deep learning (DL) are increasingly being applied in cardio-oncology to predict cardiotoxicity risk (Figure 2). Machine learning uses algorithms to identify complex patterns in structured clinical data, laboratory results, and imaging-derived metrics, capturing non-linear associations that traditional statistical models may miss. Deep learning further extends this approach by leveraging multi-layered neural networks capable of automatic feature extraction from high-dimensional data, such as CMR, echocardiography, or ECG signals, enabling detection of subtle predictors of cardiotoxicity.

Machine learning approaches based on clinical and echocardiographic variables have demonstrated promising discrimination in cardiotoxicity prediction [46]. For example, Zhou et al. developed and externally validated ML algorithms in over 4300 cancer patients using longitudinal clinical, laboratory, and echocardiographic data, achieving an AUC of 0.80 for de novo CTRCD in the derivation cohort, with consistent discrimination on temporal validation (AUC 0.79) [47]. By integrating variables such as age, cardiovascular risk factors, laboratory parameters, and LVEF, these models identified complex patterns associated with future cardiotoxicity.

In a complementary approach, DL applied to baseline ECG data has emerged as a scalable tool for risk prediction, with AI-derived signatures independently associated with subsequent cardiotoxicity [48].

Beyond structured clinical data, AI-driven analysis of cardiovascular imaging represents another promising avenue for risk prediction. A recent study by Yu et al. applied ML to pre-treatment CMR short-axis cine images in patients receiving anthracycline- and trastuzumab-based therapy [49]. The model predicted subsequent CTRCD with an AUC of 0.85 in internal validation and 0.80 in external validation, outperforming conventional clinical and manually quantified imaging models.

Despite encouraging discrimination in several studies, these findings should not be interpreted as evidence of clinical readiness. AI-based prediction tools are particularly susceptible to overfitting when model complexity is high relative to the number of outcome events, a relevant concern in cardio-oncology where CTRCD and other cardiovascular toxicities may be relatively infrequent. Additional methodological concerns include class imbalance, potential data leakage during preprocessing or feature selection, incomplete assessment of calibration, and limited independent external validation. Furthermore, the complexity and limited interpretability of some ML and DL models may hinder clinical adoption, particularly when the contribution of individual predictors cannot be readily understood. Recent methodological frameworks therefore emphasize transparent reporting, rigorous separation of development and evaluation data, calibration assessment, external validation across heterogeneous populations and settings, and evaluation of clinical utility before implementation. TRIPOD + AI provides specific guidance for transparent reporting of prediction-model studies using either regression or machine-learning methods, whereas PROBAST + AI provides a framework for assessing methodological quality, risk of bias, and applicability [50,51].

### 5.2. Digital Twins: A Future Perspective

Looking further ahead, digital twin technologies may represent a new paradigm for personalized risk prediction. A digital twin is a continuously updated computational representation of an individual patient that may combine mechanistic physiological models, such as cardiac electromechanics or hemodynamics, with longitudinal patient-specific data including clinical characteristics, treatment exposures, biomarkers, and imaging [52]. In principle, such a system could be used to simulate how an individual cardiovascular phenotype may evolve or respond to a given therapy. This differs from most conventional baseline prediction models, which provide a risk estimate at a predefined time point: a digital twin is intended to be iteratively refined as new information becomes available, potentially allowing risk estimates and simulated “what-if” scenarios to evolve throughout the course of care [53]. Digital twin approaches have already been explored in cardiovascular medicine, particularly for cardiac electrophysiology and simulation of individualized responses to arrhythmia treatment [54], but their application to cardiotoxicity prediction remains at an early stage.

In cardio-oncology, a hypothetical digital twin could be initialized using pre-treatment cardiovascular assessment and subsequently updated with cumulative anticancer treatment exposure, serial imaging findings, and circulating biomarkers. This could potentially allow simulation of individual trajectories of cardiac dysfunction and support personalized surveillance strategies. Early experimental work using MRI-based, subject-specific computational cardiac models has explored arrhythmogenic mechanisms following doxorubicin exposure, providing a proof-of-concept that is conceptually aligned with the digital-twin paradigm [55]. However, such evidence remains preclinical and does not establish the ability to predict cardiotoxicity in individual patients.

Major challenges currently limit clinical translation. Digital twins require integration of heterogeneous longitudinal data, substantial computational and modelling expertise, and rigorous validation against real-world outcomes. No widely accepted standard currently exists for validating individualized simulations at scale, and dedicated regulatory and clinical-workflow frameworks remain underdeveloped. Digital twins in cardio-oncology should therefore be considered an exploratory research direction rather than a near-term clinical tool, requiring prospective validation and demonstration of incremental clinical utility before clinical implementation.

### 5.3. Omics

Omics sciences—including genomics, proteomics, transcriptomics, and metabolomics—offer the opportunity to capture the biological heterogeneity underlying susceptibility to cancer therapy-related cardiotoxicity [56,57]. In practice, this involves profiling genetic variants, protein expression patterns, circulating microRNAs, and metabolite signatures to identify patients with increased vulnerability before the initiation of anticancer treatment. In particular, germline variants in genes involved in anthracycline metabolism, oxidative stress pathways, and DNA damage have been associated with differential risk of cardiotoxicity [58,59,60].

While genomic predictors currently represent the most developed omics-based approach for risk stratification, emerging transcriptomic studies have suggested that molecular signatures such as NFE2L2 expression may provide incremental prognostic information beyond conventional clinical variables [61].

Preliminary studies suggest that integrating multi-omics data with clinical characteristics and imaging parameters may enhance risk discrimination beyond conventional models alone, enabling a more individualized assessment of cardiotoxicity risk [62]. However, most available evidence derives from relatively small and heterogeneous cohorts, and substantial challenges—including data standardization, external validation, and demonstration of clinical utility—must be addressed before omics-based prediction can be routinely implemented in cardio-oncology practice.

## 6. Current Challenges and Limitations

### 6.1. Heterogeneity, Validation, and Transportability

Despite significant advances in cardiotoxicity risk prediction, existing approaches remain limited by substantial heterogeneity. Available tools differ substantially in intended endpoint, source population, index time, and prediction horizon, as summarized in Table 1, making direct comparison of discriminative performance potentially misleading. Models such as those by Ezaz et al. [14] and Abdel-Qadir et al. [15] estimate longer-term cardiovascular risk from pre-treatment variables, whereas RE-ACT [17] reassesses risk after anthracycline completion. Conversely, more recent CMR- and ECG-based AI approaches use a single pre-treatment acquisition to identify susceptibility to subsequent CTRCD. Prediction horizons also vary widely, from short-term detection at a single post-baseline timepoint (Yu et al. [49], Yagi et al. [48]) to long-term risk estimation over a decade (Barac et al. [16]), further limiting comparability of reported discrimination. These tools therefore address distinct clinical questions—baseline risk stratification, post-treatment reassessment, and pre-treatment susceptibility—which cannot be reconciled through direct comparison of AUC values alone.

A second major source of heterogeneity is the definition of the predicted outcome. Cardiotoxicity has been operationalized across studies as symptomatic heart failure or cardiomyopathy, asymptomatic LVEF decline, broader MACE composites, or heterogeneous post-treatment cardiovascular events. RE-ACT, for example, defines cardiotoxicity as either a >10 percentage-point LVEF decline to <50% or symptomatic heart failure, whereas Yagi et al. used an LVEF-based CTRCD definition. Barac et al., by contrast, identified HF/cardiomyopathy events using administrative diagnostic codes rather than the consensus CTRCD definition, due to the lack of systematic echocardiographic data—likely capturing overt events while missing milder or subclinical dysfunction. Such differences substantially affect event incidence and clinical relevance, limiting direct interchangeability between approaches even when their reported discrimination appears similar.

Competing events further complicate long-term risk prediction in oncology. Abdel-Qadir et al. explicitly accounted for non-cardiovascular mortality using competing-risk methodology, whereas this has not been consistently incorporated across available tools. Failure to account for competing mortality may distort absolute risk estimates and calibration, particularly in older patients or populations with substantial cancer-related mortality. More broadly, attenuation of model performance during external validation may reflect differences in case mix, treatment exposure, endpoint ascertainment, and baseline risk between derivation and validation populations. This is illustrated by the poor external discrimination reported for the Ezaz model (AUC 0.51) when applied to a substantially younger contemporary HER2-positive breast cancer cohort [9].

Taken together, heterogeneity across cardiotoxicity risk prediction approaches reflects not only methodological limitations but also fundamental differences in what is being predicted, in whom, and at what point in the cancer treatment trajectory. Harmonized outcome definitions, prespecified prediction horizons, appropriate handling of competing risks, and robust external validation are therefore essential before meaningful cross-model comparison and clinical implementation.

### 6.2. Clinical Utility

Beyond statistical performance, the clinical impact of existing approaches has been insufficiently evaluated. Most studies focus on discrimination and calibration, with few assessing net clinical benefit, cost-effectiveness, or changes in decision-making [3]. As a result, it remains unclear whether these tools improve patient management or reduce unnecessary investigations. Current guideline recommendations still rely on uniform surveillance strategies rather than individualized risk, potentially leading to over-screening, repeated imaging, patient anxiety, and increased healthcare resource utilization without demonstrable improvement in outcomes.

### 6.3. Applicability to Contemporary Therapies and Vulnerable Patient Populations

Most available risk-prediction tools were developed and validated in populations treated with anthracyclines and anti-HER2 agents, whose cardiotoxicity is typically dose-dependent and cumulative for anthracyclines, and mechanistically linked to interference with cardiomyocyte survival pathways for HER2-targeted therapies [4]. Newer classes of cancer therapy are associated with fundamentally different cardiotoxic mechanisms, raising the question of whether existing prediction paradigms are conceptually transferable rather than merely under-validated [63].

ICI-associated cardiotoxicity, most notably myocarditis, is immune-mediated rather than dose-dependent, can occur early after treatment initiation, and follows an unpredictable and potentially fulminant course unrelated to cumulative exposure [64]. Traditional cardiovascular risk factors and baseline LVEF—the backbone of most statistical models in Table 1—have shown limited ability to identify patients at risk of ICI-related myocarditis, and no validated risk-prediction tool is currently available for this setting; emerging candidate predictors include pre-existing autoimmune conditions and combination immunotherapy regimens, but these remain investigational [65].

Antibody–drug conjugates (ADCs) present a further layer of heterogeneity, as cardiovascular toxicity may depend on multiple drug-specific features, including the antibody target, linker characteristics, cytotoxic payload, and off-target exposure [66]. Consequently, toxicity patterns may differ substantially across individual ADCs, limiting the generalizability of risk-prediction approaches from one agent to another. Cardiotoxicity data for bispecific antibodies and cellular therapies such as CAR-T cells are even more limited and are further complicated by their association with cytokine release syndrome (CRS), which can independently produce arrhythmias, hypotension, and reversible myocardial dysfunction through an acute inflammatory mechanism distinct from the chronic, structural damage targeted by conventional cardiotoxicity risk scores [67]. Prediction in this setting may require integration of inflammatory and tumor-burden-related variables rather than conventional cardiovascular risk factors alone [68].

Similarly, tyrosine kinase inhibitors encompass heterogeneous cardiovascular toxicity profiles—including hypertension, vascular events, QT prolongation, and myocardial dysfunction—which vary according to the molecular target and individual agent, making a single shared prediction framework unlikely to be applicable across the entire class [69].

Vulnerable populations—including elderly patients, those with multiple comorbidities, and patients with pre-existing cardiovascular disease—are similarly underrepresented across nearly all models summarized in Table 1: several explicitly excluded patients with baseline cardiac dysfunction or restricted enrollment to younger age ranges. This is a consequential gap, as these patients face a higher competing risk of non-cardiovascular mortality and a greater potential benefit from accurate risk stratification, yet are the least represented in model derivation cohorts. Several prospective collaborative initiatives, such as CARDIOCARE (An Interdisciplinary Approach for the Management of the Elderly Multimorbid Patient with Breast Cancer Therapy Induced Cardiac Toxicity), are emerging to address these gaps by integrating multicenter clinical data, imaging, biomarkers, and AI-driven predictive models, aiming to improve individualized risk stratification and surveillance [70].

Taken together, extending risk prediction to these settings will likely require dedicated models incorporating therapy-specific mechanisms and dynamic, treatment-phase-aware variables, rather than direct extrapolation from tools derived in the anthracycline/anti-HER2 setting.

**Table 1:** Comparative overview of cardiotoxicity risk prediction approaches. CV, cardiovascular; CTRCD, cancer therapy-related cardiac dysfunction; HF, heart failure; MACE, major adverse cardiovascular events; LVEF, left ventricular ejection fraction; HER2, human epidermal growth factor receptor 2; AUC, area under the curve; AUROC, area under the receiver operating characteristic curve; C-index, concordance index; D, derivation cohort; EV, external validation; IV, internal validation; ML, machine learning; AI-ECG, artificial intelligence-enabled electrocardiography; ML-CMR, machine learning-based cardiac magnetic resonance; y, years; HL, Hosmer–Lemeshow.

Model/Framework	Type	Population/Cancer Therapy	Predicted Endpoint	Derivation Cohort (N)	External Validation	Discriminative Performance	Calibration	Main Limitations
**Mayo Clinic risk score** [8]	Consensus framework	General oncology population	Broad cardiotoxicity risk	None—expert consensus	Yes [71]	AUC EV 0.654 for CTRCD [71]	N/A at development; not reported in EV	Not data-driven
**HFA-ICOS** [5]	Consensus framework	Therapy-specific proformas	Therapy-specific cardiotoxicity risk	None—expert consensus	Yes, several cohorts [9,10,11,12,17,72,73]	Variable across studies	Variable in EV (poor-good; Brier 0.04 in one cohort)	Categorical, not continuous; inconsistent external validation results
**Ezaz et al.** [14]	Statistical model	Older women, HER2+ breast cancer, trastuzumab	HF/cardiomyopathy at 3 y	1664	Yes [9,74,75]	AUC EV 0.51 for CTRCD [9]	HL *p* = 0.76; poor in EV	Restricted population
**Abdel-Qadir et al.** [15]	Statistical model	Early-stage breast cancer	MACE at 5 and 10 y	90,104	Yes [76]	c-index IV 0.82–0.80; EV 0.62–0.66 for MACE at 5–10 y [15,76]	Good at 5–10 y; poor in EV	Breast cancer specific
**Barac et al.** [16]	Statistical model	Breast cancer	HF/cardiomyopathy at 10 y	26,044	No	c-index D 0.75; IV 0.76	Good at 5–10 y in D and IV (HL *p* > 0.05)	Breast cancer specific
**RE-ACT** [17]	Statistical model	Anthracycline-treated adult patients	Post-treatment HF/LVEF decline	2612	Yes, external cohort in original study	AUC D 0.83; EV 0.88	Good graphical calibration at 1 y in D and EV	Anthracycline-specific
**Zhou et al.** [47]	ML algorithm	Mixed oncology population	De novo CTRCD	4309	No	AUROC D 0.80; IV 0.79	Not reported	Limited clinical implementation
**Yagi et al**. [48]	AI-ECG	Mixed oncology population	LVEF decline	1328	No	AUC D 0.78 at 2 y	Not reported	Limited clinical implementation
**Yu et al.** [49]	DL-CMR	Breast cancer, anthracycline/HER2 inhibitors	De novo CTRCD	229	Yes, external cohort in original study	AUC D 0.85; EV 0.80	Not reported	Imaging-intensive; limited generalizability

## 7. Conclusions

Cardiotoxicity risk prediction in oncology is progressively evolving from static, baseline-driven approaches toward more integrative approaches incorporating imaging, biomarkers, and computational methods. Despite substantial methodological advances, currently available tools remain limited in their ability to capture the dynamic, treatment-dependent, and biologically heterogeneous nature of cardiovascular toxicity in cancer patients. As a result, no existing strategy provides sufficiently precise or adaptable risk estimation to fully support individualized clinical decision-making. Future progress will depend on the development of longitudinal, outcome-driven frameworks that integrate multimodal data within continuously updated predictive systems, moving beyond single-time-point assessment toward truly dynamic risk modelling in cardio-oncology.

## Data Availability

No new data were created or analyzed in this study. Data sharing is not applicable to this article.

## Abbreviations

The following abbreviations are used in this manuscript:

AI	Artificial Intelligence
AUC	Area Under the Curve
CAC	Coronary Artery Calcium
CMR	Cardiac Magnetic Resonance
CT	Computed Tomography
CTRCD	Cancer Therapy-Related Cardiac Dysfunction
DL	Deep Learning
DNA	Deoxyribonucleic Acid
ECG	Electrocardiogram
GLS	Global Longitudinal Strain
HER2	Human Epidermal Growth Factor Receptor 2
HFA-ICOS	Heart Failure Association–International Cardio-Oncology Society
LVEF	Left Ventricular Ejection Fraction
MACE	Major Adverse Cardiovascular Events
ML	Machine Learning
RV	Right Ventricle
SEER	Surveillance, Epidemiology, and End Results Program
